# Identification of Two CDK5R1-Related Subtypes and Characterization of Immune Infiltrates in Alzheimer's Disease Based on an Integrated Bioinformatics Analysis

**DOI:** 10.1155/2022/6766460

**Published:** 2022-12-13

**Authors:** Xu Shao, Yanxian Yang, Jieyun Chen, Runping Zhao, Lei Xu, Xilong Guo, Yu Feng, Lina Qin

**Affiliations:** ^1^Zhongshan School of Medicine, Sun Yat-sen University, Guangzhou, China; ^2^Chengdu Medical College, Chengdu, China; ^3^Department of Histology and Embryology, Zhongshan School of Medicine, Sun Yat-sen University, Guangzhou 51008, China

## Abstract

**Background:**

Alzheimer's disease (AD) is a neurodegenerative disorder and the major cause of senile dementia. The Reelin pathway has been involved in both learning and AD pathogenesis. However, the specific Reelin-related gene signature during the pathological process remains unknown.

**Methods:**

Reelin-related gene (CDK5R1) expression was analyzed using the GEO datasets. The relevant genes of CDK5R1 were identified using differential expression analysis and weighted gene correlation network analysis (WGCNA) based on the GSE43850 dataset. ConsensusClusterPlus analysis was applied to identify subtypes (C1 and C2) of AD. The CIBERSORT algorithm was used to assess the immune cell infiltration between the two AD subtypes.

**Results:**

CDK5R1 was downregulated in AD. 244 differentially expressed CDK5R1-related genes (DECRGs) between the two subgroups were mainly enriched in GABAergic synapse, neuroactive ligand-receptor interaction, synapse organization, neurotransmitter transport, etc. Furthermore, the GSVA results indicated that immune-related pathways were significantly enriched in the C1 subgroup. Interestingly, 10 Reelin pathway-related genes (CRK, DAB2IP, LRP8, RELN, STAT5A, CDK5, CDK5R1, DAB1, FYN, and SH3KBP1) were abnormally expressed between the two subgroups. The proportion of T cell gamma delta, monocytes, macrophage M2, and dendritic cells activated decreased from C1 to C2, while the proportion of plasma cells, T cell follicular helper, and NK cells activated increased.

**Conclusion:**

Two CDK5R1-related subtypes of AD were identified, helping us to better understand the role of CDK5R1 in the pathological process of AD.

## 1. Introduction

Alzheimer's disease (AD) is a common form of neurodegenerative dementia with a progressive deficit of cognitive functions, such as social disorders, behavioral abnormalities, cognitive dysfunction, and memory loss [1, 2]. The incidence of AD is positively correlated with age, with about 20% of AD patients over 65 years of age [3]. According to statistics, nearly half a million new cases of AD are recorded each year, and AD is the fifth leading cause of death for people 65 and older [4, 5]. However, due to the complex pathological mechanism of AD, most treatment strategies cannot successfully prevent or cure AD. Therefore, the identification of molecular markers to understand the etiology and pathogenesis of AD is of great significance for the early diagnosis, prevention, and treatment of AD.

Various hypotheses have been proposed regarding the pathological mechanism of AD. Based on previous studies, the abnormal hyperphosphorylation of tau protein, neuroinflammation, mitochondrial cascade, oxidative stress, and deposition of amyloid *β*-protein are the primary pathogeneses of AD [1, 6, 7]. The Reelin signaling pathway was involved in the pathogenesis of human brain diseases, including epilepsy, AD, mental retardation, depression, bipolar disorder, schizophrenia, autism, and lissencephaly [8]. The changes in Reelin signaling or processing were associated with AD-related neuronal dysfunction [9]. Cyclin-dependent kinase 5 regulatory subunit 1 (CDK5R1) is one of the key genes in the Reelin pathway, whose activity plays an important role in neuronal differentiation and migration during neurodevelopment and is involved in neurodegenerative diseases [10, 11]. Suppression of CDK5R1 activity improved diabetes-related cognitive deficits [12]. The polymorphisms and mutations in CDK5R1 and CDK5 contributed to the onset of intellectual disability [13]. The miR-15/107 family plays an important role in the pathogenesis of AD through the upregulation of CDK5R1/p35 levels [14]. It has been reported that CDK5R1 is an important regulator participating in the aberrant hyperphosphorylation of tau in AD [15]. These studies implied that CDK5R1 has a vital role in AD. However, the CDK5R1 expression differences that existed in AD patients have not been investigated.

**Figure 1:**
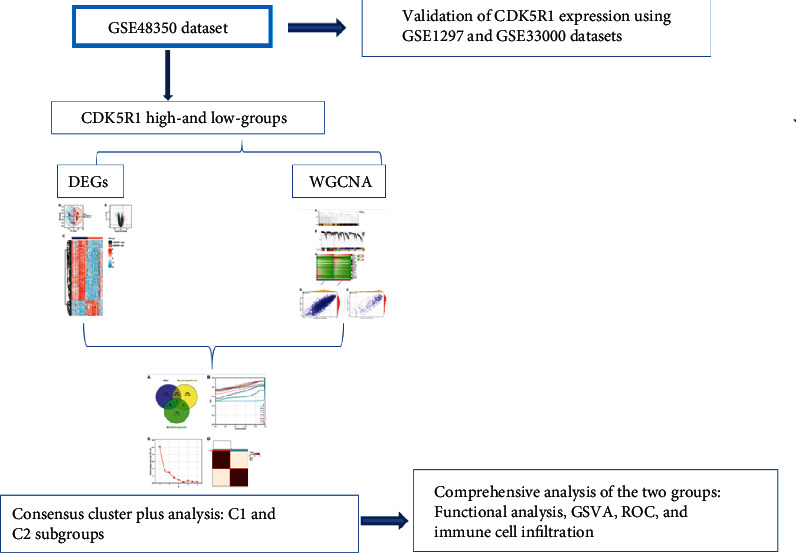
The flowchart of this study.

The rapid development of bioinformatics technology provides a powerful technical mean for exploring new therapeutic targets and complex disease mechanisms [16–20]. In the present study, we aimed to identify CDK5R1-related gene signatures and CDK5R1-related AD subtypes that are implicated in AD pathogenesis via integrated bioinformatics analysis. The flowchart of the present study is shown in Figure 1. Our research will provide a novel perspective for further understanding the CDK5R1 implicated in AD development.

## 2. Methods

We acquired the AD transcriptome data sets (GSE48350, GSE1297, and GSE33000) from Gene Expression Omnibus (GEO) database. The GSE48350 dataset contains 173 normal samples and 80 AD samples, with the platform GPL570. The GSE1297 dataset includes 9 normal samples and 7 severe AD samples, with the platform GPL96. The GSE33000 dataset contains 157 normal samples and 310 AD samples, with the platform GPL4372. Detailed information on these datasets is shown in Table 1. We downloaded the raw data of these datasets using the “GEOquery” package. And the “justRMA” function from the “affy” package was used for the normalization of these gene expression profiles. Reelin pathway-associated genes were obtained from the MSigDB database.

### 2.1. Identification of CDK5R1-Related Differentially Expressed Genes (DEGs)

80 AD samples from the GSE48350 dataset were divided into low- and high-CDK5R1 subgroups based on the median expression of CDK5R1. Then, we used the limma R package of R to identify DEGs between the two subgroups by setting adjusted *p* < 0.05 and |logFC| ≥ 1 [21]. The heat map and volcano plots of these DEGs were visualized using “pheatmap” and “limma” packages of R [22, 23].

### 2.2. Weighted Gene Correlation Network Analysis (WGCNA)

The “WGCNA” package of R was used to perform WGCNA based on the gene expression profiles of the GSE48350 dataset (80 AD samples) [24]. The “pickSoftThreshold” function of the WGCNA package was applied to calculate the soft threshold. A topological overlap matrix (TOM) was constructed by transforming the adjacency matrix. We used the dynamic tree cut to identify the coexpressed gene modules in the low- and high-CDK5R1 subgroups. The important modules with the highest correlation genes were selected for further analysis. Mode membership (MM) > 0.8 and gene significance (GS) > 0.5 were considered the threshold to identify hub genes in the key modules [25].

### 2.3. Consensus Clustering of Subtypes Based on CDK5R1-Related Genes in AD Patients

We used the “ConsensusClusterPlus” R package to investigate the expression pattern of the CDK5R1-related DEGs in AD patients. We performed consensus clustering using the *k*-means algorithm with repeat 100 times of 80% of the total samples.

### 2.4. Identification and Analysis of Differentially Expressed CDK5R1-Related Genes (DECRGs) in the Two Subtypes

The limma R package was used to identify DECRGs between the C1 and C2 subgroups by setting adjusted *p* < 0.05 and |logFC| ≥ 1.5. The heat map and volcano plots of these DECRGs were visualized using “pheatmap” and “limma” packages of R. The Gene Ontology (GO) and Kyoto Encyclopedia of Genes and Genomes (KEGG) analyses were carried out using the “clusterProfiler” package to investigate the potential pathways of DECRGs. *p* < 0.05 was considered statistically significant. Besides, we also used the “GSVA” R package to perform the gene set variation analysis (GSVA) for the investigation of signaling pathway change between the C1 and C2 subgroups.

### 2.5. Immune Analyses

To analyze the immune cell infiltration levels between the two subgroups, we used the Cell-type Identification by Estimating Relative Subsets of RNA Transcripts (CIBERSORT) algorithm to assess the immune infiltration. ComplexHeatmap package of R was applied to visualize the infiltration level of immune cells. The immune cell infiltration levels between the C1 and C2 subgroups were visualized via drawing boxplots using the “ggplot2” package of R.

### 2.6. Statistical Analysis

The R software (v4.0.3) was used to perform the data statistical analysis. The differences between the two groups were analyzed using the Wilcoxon rank sum test. Statistical significance was set at *p* < 0.05.

## 3. Results

### 3.1. Expression of CDK5R1 in AD

**Figure 2:**
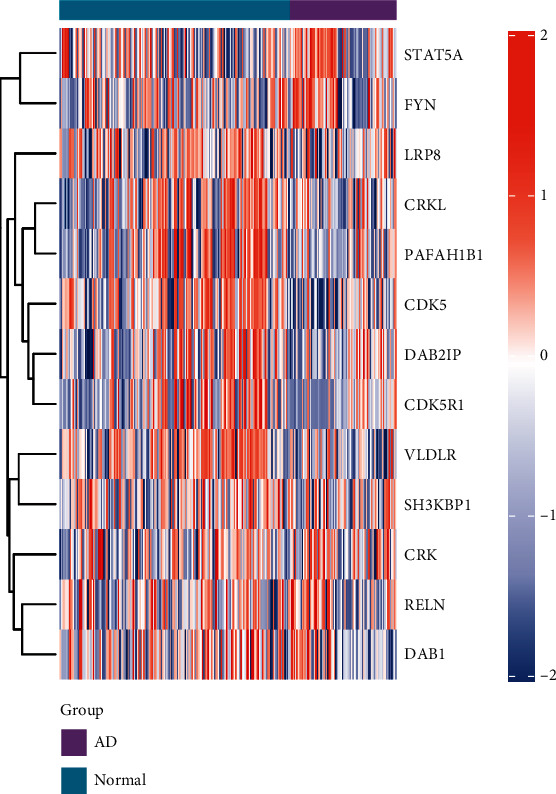
The heat map of Reelin pathway-related genes in the GSE48350 dataset. Red indicates upregulation, while light blue indicates downregulation.

**Figure 3:**
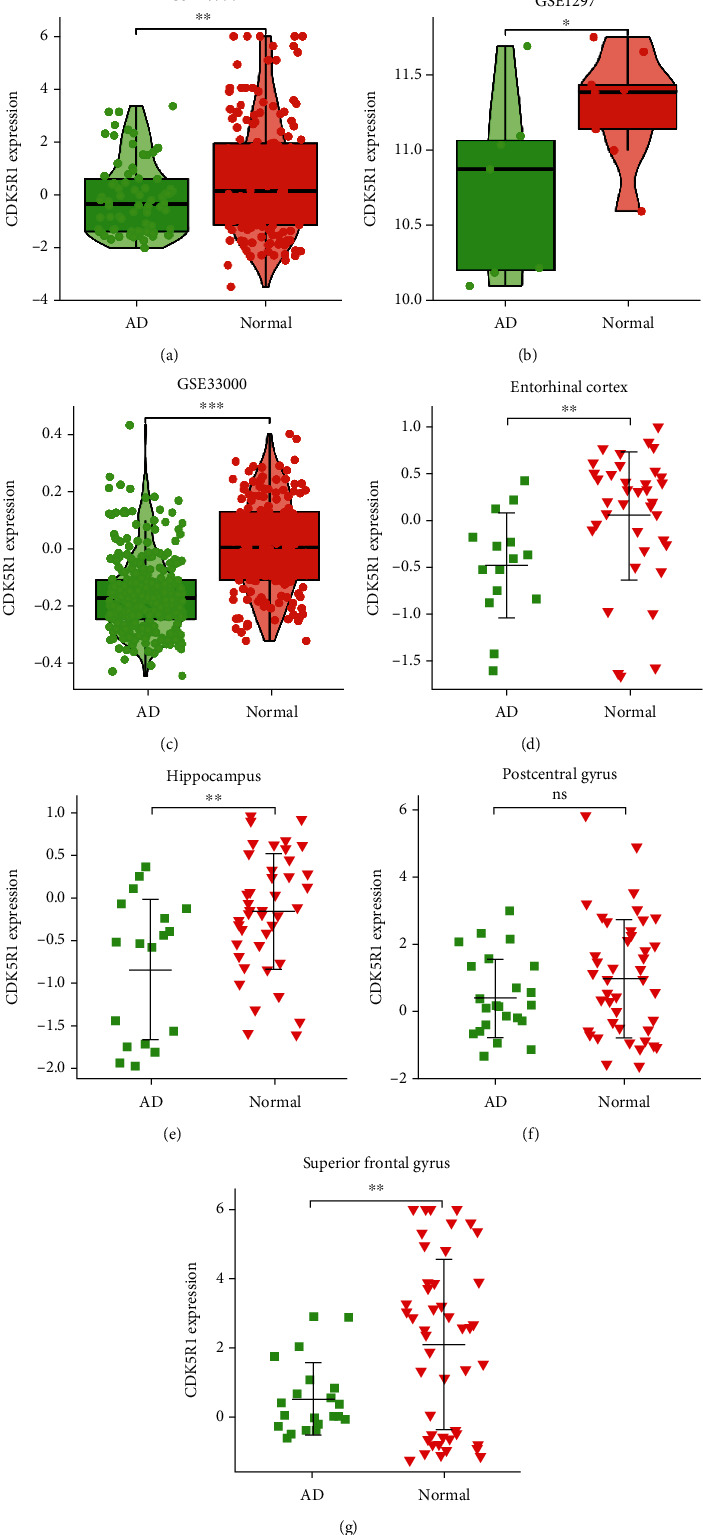
The CDK5R1 expression in the GEO database. Analysis of CDK5R1 expression levels between the AD and normal groups based on GSE48350 (a), GSE1297 (b), and GSE33000 (c) datasets. CDK5R1 expression in the entorhinal cortex (d), hippocampus (e), postcentral gyrus (f), and superior frontal gyrus (g) in the GSE48350 dataset.

Firstly, we analyzed 13 Reelin pathway-related genes in GSE48350. The expression of CDK5R1 in AD samples was lower than that of normal samples (Figures 2 and 3(a)). We also analyzed the CDK5R1 expression levels in GSE1297 and GSE33000 datasets (Figures 3(b) and 3(c)), and the results showed that CDK5R1 was significantly downregulated in AD patients. Furthermore, we further analyzed the CDK5R1 expression levels in different brain regions in the GSE48350 dataset, due to the heterogeneity of brain tissue. As shown in Figures 3(d)–3(g), we found that CDK5R1 expression was downregulated in the entorhinal cortex, hippocampus, and superior frontal gyrus in AD samples compared to normal samples. However, there was no significant difference in CDK5R1 level in the postcentral gyrus (Figure 3(f)). These findings showed that CDK5R1 was abnormally expressed in AD patients, implying that CDK5R1 may play an important role in AD pathogenesis.

### 3.2. Identification of DEGs

**Figure 4:**
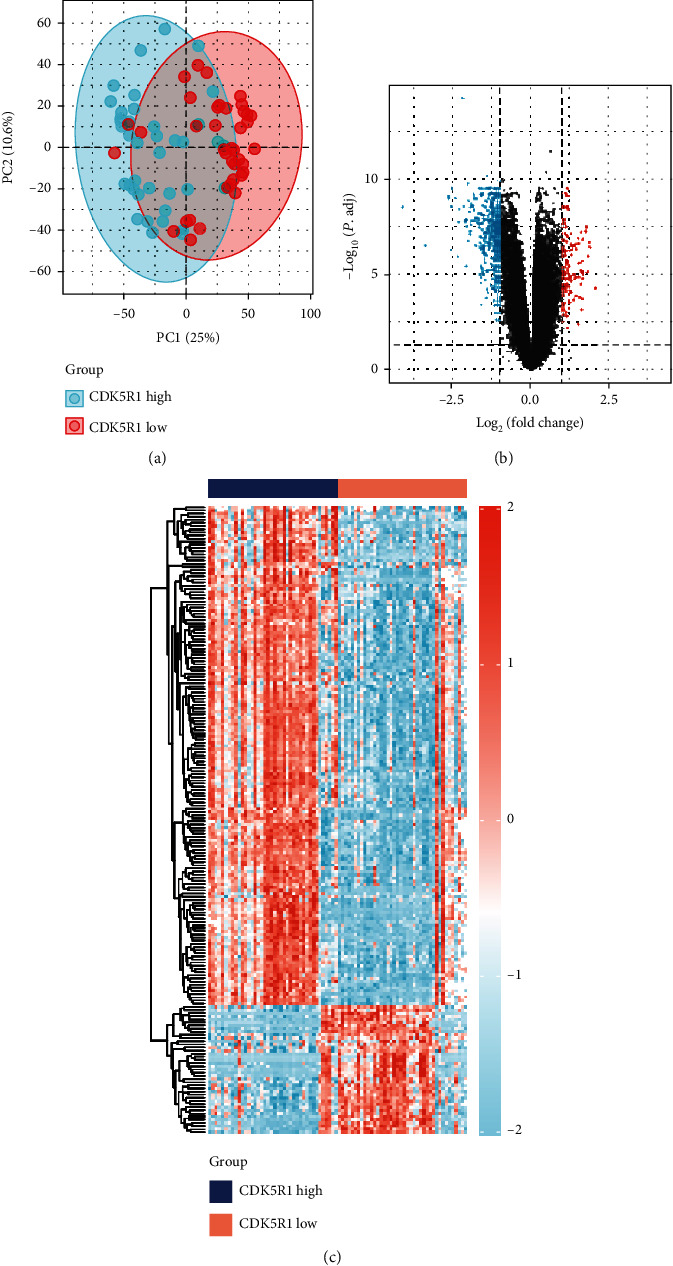
Identification of DEGs. (a) PCA of the high- and low-CDK5R1 expression groups after standardization. (b) The volcano plot presented the DEGs in the high- and low-CDK5R1 expression groups. The blue dots indicate downregulated genes; the red dots indicate upregulated genes. (c) Heat map presented the DEGs in the high- and low-CDK5R1 expression groups. Red indicates upregulation, while light green indicates downregulation.

The AD samples of the GSE48350 dataset were divided into CDK5R1 low- and high-expression groups based on the median level of CDK5R1. The PCA result indicated that there were some differences between the CDK5R1 low-expression and CDK5R1 high-expression groups (Figure 4(a)). A total of 441 DEGs were significantly expressed between the two groups (Figure 4(b)). Among them, 334 DEGs were downregulated and 107 DEGs were upregulated in the CDK5R1 low-expression group compared with those in the CDK5R1 high-expression group (Figure 4(c)).

### 3.3. Identification of Key Modules Associated with CDK5R1 in AD

**Figure 5:**
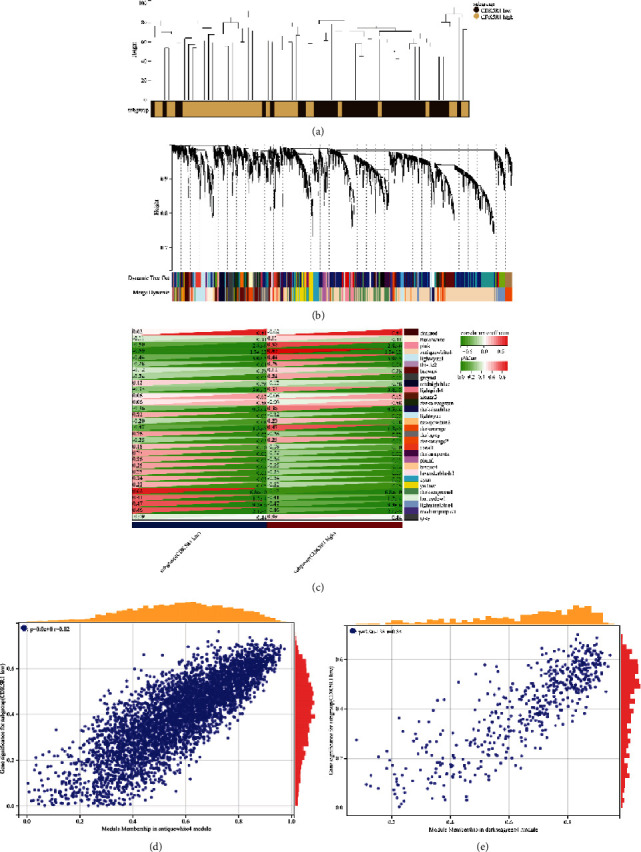
Construction of coexpression modules related to CDK5R1 in AD. (a) Clustering dendrogram of 80 AD samples. (b) In the cluster dendrogram of genes in the GSE48350 dataset, all genes were clustered in 30 modules. (c) Module-trait relationship of two traits and 30 modules. The scatter plot presented the correlation between gene significance and module membership in the antiquewhite4 module (d) and darkseagreen4 module (e).

We performed WGCNA to identify the key modules related to CDK5R1 in AD. After merging similar modules, we identified a total of 30 modules in the two subgroups (Figures 5(a) and 5(b)). We drew a heat map to present the correlated modules (Figure 5(c)), and the results showed that the antiquewhite4 module exhibited the strongest positive correlation with CDK5R1 (*p* = 1.3*e* − 12, *r* = 0.69), whereas the darkseagreen4 module exhibited the strongest negative correlation with CDK5R1 (*p* = 6.8*e* − 10, *r* = −0.62). Therefore, the two modules were selected for the following analysis by setting the thresholds of MM > 0.8 and GS > 0.5 (Figures 5(d) and 5(e)).

### 3.4. Identification of CDK5R1 Subgroups Using Consensus Clustering

**Figure 6:**
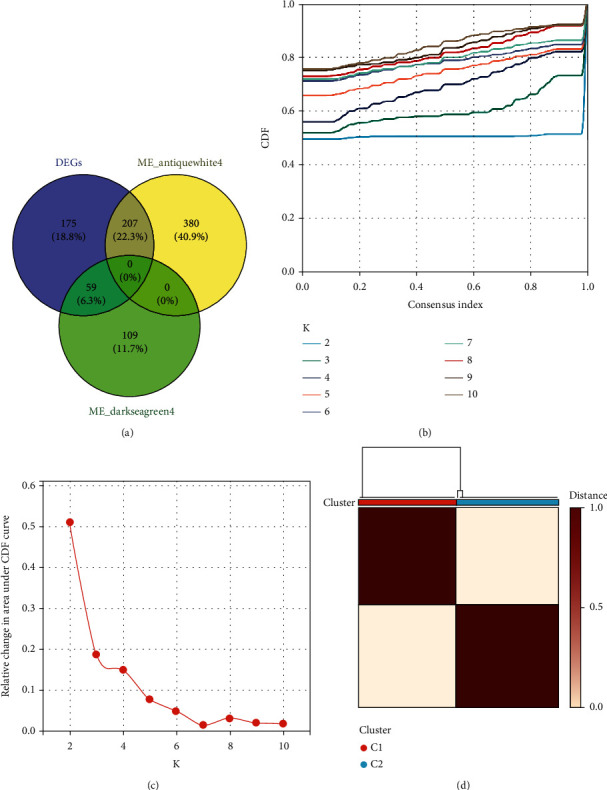
Construction of CDK5R1-related gene clusters. (a) The common genes between DEGs and key modules. (b) The cumulative distribution function (CDF) curve for *k* = 2‐10. (c) Delta area map. (d) The matrix heat map indicates the consensus matrix at *k* = 2 in the GSE48350 dataset.

A total of 207 intersection genes between the DEGs and antiquewhite4 module were obtained, and 59 intersection genes between the DEGs and darkseagreen4 module were obtained. Then, a total of 266 common genes were used to carry out consensus clustering (Figure 6(a)). The 80 AD samples of GSE48350 were clustered into two CDK5R1-related subtypes based on these 266 common genes. Based on the CDF curves (Figure 6(b)) and delta area map (Figure 6(c)), we selected the optimal division (*k* = 2) as the optimal number of clusters. Therefore, the 80 AD samples were divided into C1 (*n* = 39) and C2 (*n* = 41) subgroups (Figure 6(d)).

### 3.5. Identification and Analysis of DECRGs in the C1 and C2 Subtypes

**Figure 7:**
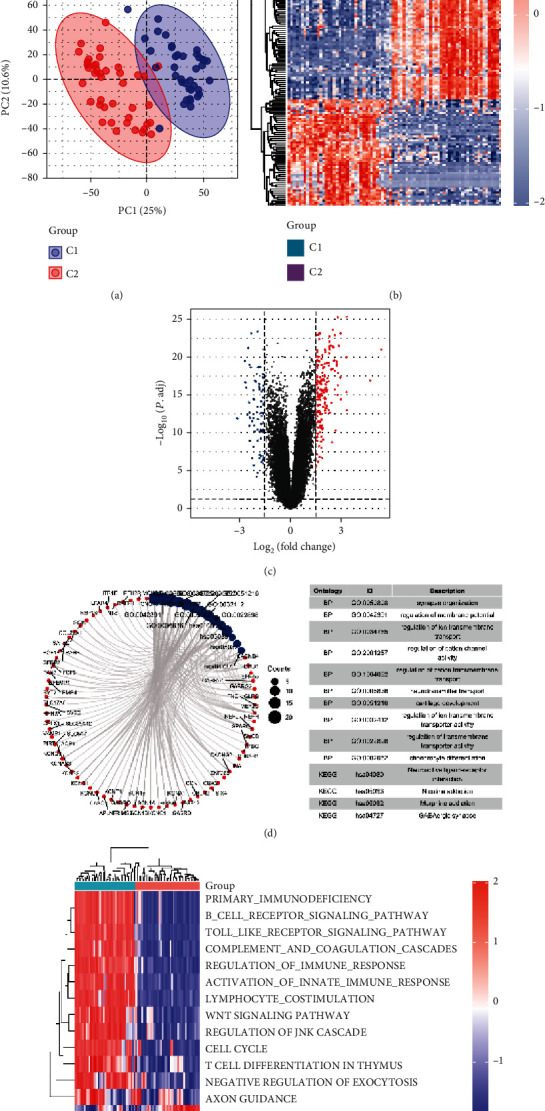
Identification of DECRGs. (a) PCA of the C1 and C2 subgroups after standardization. (b) Heat map presented the DECRGs in the C1 and C2 subgroups. Red indicates upregulation, while light blue indicates downregulation. (c) The volcano plot presented the DECRGs in the C1 and C2 subgroups. The blue dots indicate downregulated genes; the red dots indicate upregulated genes. (d) Functional enrichment analysis of the DECRGs. The red dots indicate genes; the blue dots indicate enrichment pathways. (e) Heat map showed the activation state of potential pathways in the C1 and C2 subgroups after processing using GSVA. Blue indicates inactivation, while red indicates activation.

The PCA result indicated significant differences between the C1 and C2 subgroups (Figure 7(a)). A total of 244 DECRGs were significantly expressed between the two groups (Figure 7(b)). Among them, 79 DECRGs were downregulated and 165 DECRGs were upregulated in the C2 group compared with those in the C1 group (Figure 7(c)).

We performed the functional enrichment analyses of 244 DECRGs. As shown in Figure 7(d) and Table 2, the primary enrichment pathways for DECRGs were the synapse organization, regulation of cation channel activity, neurotransmitter transport, regulation of transmembrane transporter activity, neuroactive ligand-receptor interaction, and GABAergic synapse. Furthermore, we also performed GSVA to explore the potential biological pathways enriched in the C1 and C2 subgroups. Our findings indicated that CDK5R1-related genes were mainly enriched in primary immunodeficiency, B cell receptor signaling pathway, Toll-like receptor signaling pathway, complement and coagulation cascades, regulation of immune response, activation of the innate immune response, lymphocyte costimulation, WNT signaling pathway, regulation of JNK cascade, cell cycle, T cell differentiation in the thymus, negative regulation of exocytosis, and axon guidance (Figure 7(e)), and these pathways were inhibited in the C2 subgroup.

### 3.6. Expression Levels and Diagnostic Value of Reelin Pathway-Related Genes

**Figure 8:**
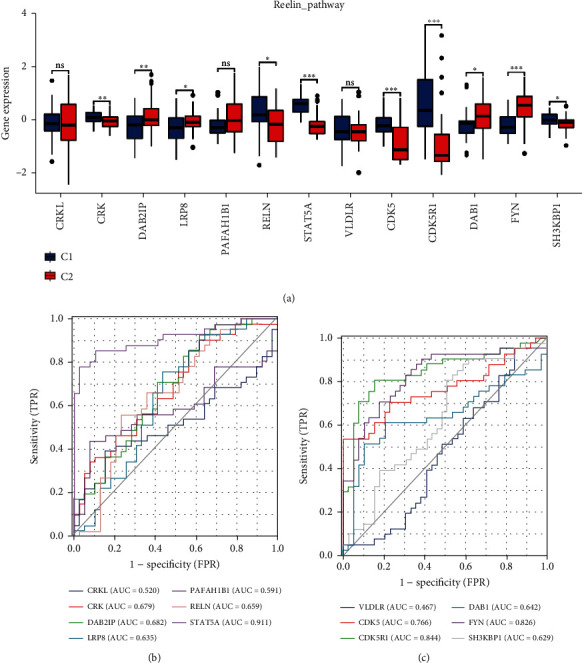
Expression levels (a) and a diagnostic value (b, c) of Reelin pathway-related genes in the C1 (*n* = 39) and C2 (*n* = 41) subgroups. Subtypes were compared using the Wilcoxon rank sum test. ^∗^*p* < 0.05, ^∗∗^*p* < 0.01, and ^∗∗∗^*p* < 0.001.

We compared the expression levels of Reelin pathway-related genes between the C1 and C2 subgroups. As shown in Figure 8(a), CRK, RELN, STAT5A, CDK5, CDK5R1, and SH3KBP1 were downregulated, whereas DAB2IP, LRP8, DAB1, and FYN were upregulated in the C2 group compared with those in the C1 group. Besides, we also assessed the diagnostic values of these genes in the two subgroups, and findings revealed that the diagnostic AUC values of STAT5A, CDK5, CDK5R1, and FYN genes were 0.911, 0.766, 0.844, and 0.826, respectively (Figures 8(b) and 8(c)). Our results indicated that these genes had high diagnostic ability for distinguishing AD subgroups.

### 3.7. Immune Characteristics of the CDK5R1-Related Subtypes in AD

**Figure 9:**
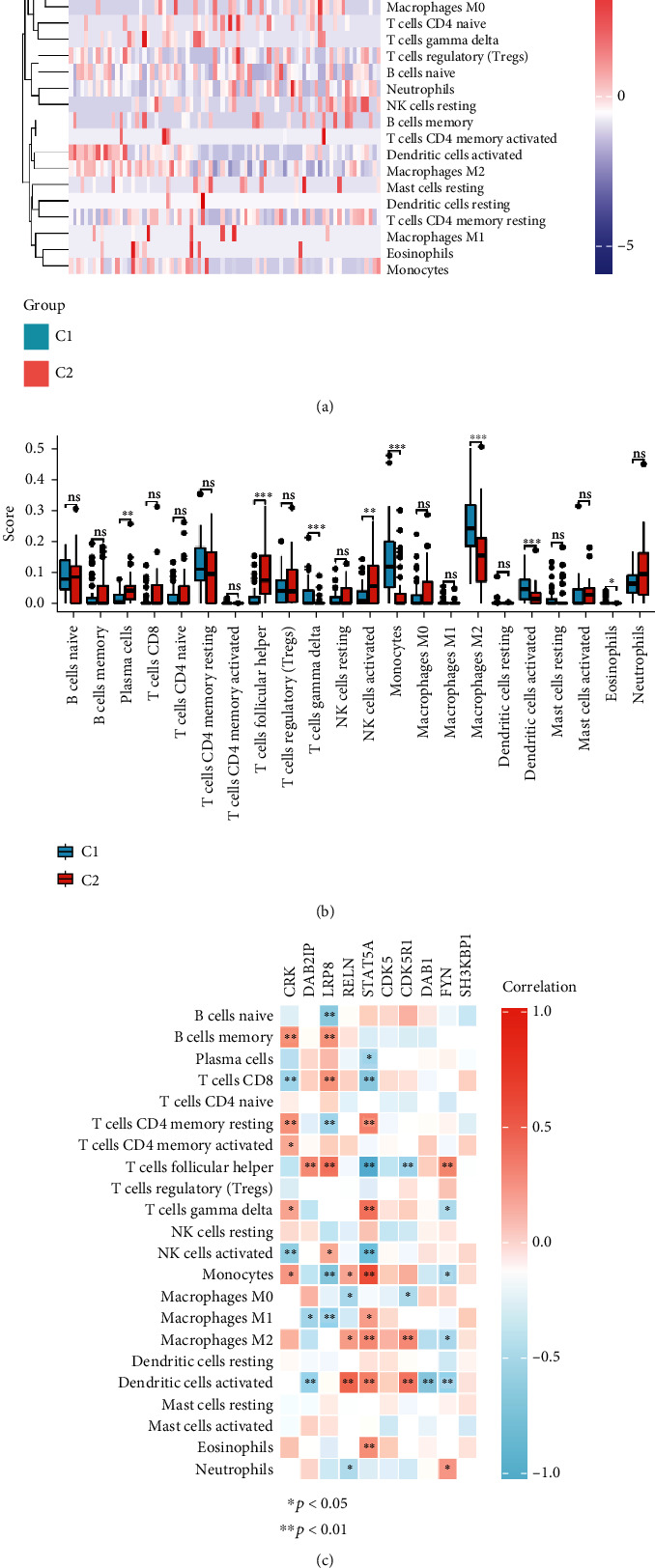
Immune characteristics of the CDK5R1-related subtypes in AD. (a) Heat map of 22 types of immune cells in the two subgroups. (b) The immune cell infiltration levels between C1 and C2 subgroups. (c) The correlation of CRK, DAB2IP, LRP8, RELN, STAT5A, CDK5, CDK5R1, DAB1, FYN, and SH3KBP1 expressions with 22 types of immune cells in AD.

In this study, we used the CIBERSORT algorithm to further assess the immune response of AD patients. As shown in Figure 9(a), the heat map of 22 types of immune cell infiltration levels in AD samples indicated that plasma cells, T cell follicular helper, T cell gamma delta, NK cells activated, monocytes, and macrophage M2 were significantly different between the C1 and C2 subgroups. Besides, the proportions of plasma cells, T cell follicular helper, and NK cells were significantly lower in the C1 subgroup than the C2 subgroup, whereas the proportions of monocytes, macrophage M2, T cell gamma delta, and dendritic cells activated were higher in the C1 subgroup than the C2 subgroup (Figure 9(b)). Based on the results of correlation analysis, the CRK expression was positively correlated with B cell memory, T cell CD4 memory resting, T cell CD4 memory activated, T cell gamma delta, and monocytes whereas negatively correlated with T cells CD8 and NK cells activated; LRP8 was positively correlated with B cell memory, T cell CD8, T cell follicular helper, and NK cells activated whereas negatively correlated with B cell naïve, T cell CD4 memory resting, monocytes, and macrophage M1; DAB2IP expression was positively correlated with T cell follicular helper whereas negatively correlated with macrophage M1 and dendritic cells activated; RELN was positively correlated with monocytes, macrophage M2, and dendritic cells activated whereas negatively correlated with macrophage M0 and neutrophils; STAT5A was positively correlated with T cell CD4 memory resting, T cell gamma delta, monocytes, macrophage M1, macrophage M2, and dendritic cells activated whereas negatively correlated with plasma cells, T cells CD8, T cells follicular helper, and NK cells activated; CDK5R1 was positively correlated with macrophage M2 and dendritic cells activated whereas negatively correlated with T cell follicular helper and macrophage M0; DAB1 was negatively correlated with dendritic cells activated; FYN was positively correlated with T cell follicular helper and neutrophils whereas negatively correlated with T cell gamma delta, monocytes, macrophage M2, and dendritic cells activated (Figure 9(c)).

## 4. Discussion

AD is a degenerative disease of the central nervous system that occurs in old age. The pathological mechanism of AD is not clear, and there is no radical cure at present [26]. A large number of studies have indicated that the pathological development of AD preceded the appearance of clinical symptoms by several decades [27]. Thus, the identification of potential biomarkers will contribute to the early diagnosis of AD and provide potential therapeutic targets for its treatment. CDK5R1 plays an important role in the central nervous system development [28]. In the present study, we found that CDK5R1 is downregulated in AD patients, which implied its potential role in AD development. Besides, our findings also provided a scientific basis for effective diagnosis and individual treatment of AD.

Ten Reelin pathway-related genes (CRK, DAB2IP, LRP8, RELN, STAT5A, CDK5, CDK5R1, DAB1, FYN, and SH3KBP1) may serve as potential diagnostic markers for AD patients. We found that the AD patients could be divided into two subgroups (C1 and C2) using a series of bioinformatics analyses, such as WGCNA and ConsensusClusterPlus analysis. GSVA revealed that the AD patients in the C1 subgroup were mainly enriched with DECRGs related to the primary immunodeficiency, B cell receptor signaling pathway, Toll-like receptor signaling pathway, complement and coagulation cascades, regulation of immune response, activation of innate immune response, lymphocyte costimulation, WNT signaling pathway, regulation of JNK cascade, cell cycle, T cell differentiation in thymus, negative regulation of exocytosis, and axon guidance. Previous studies have revealed that CRK plays specific roles in regulating immune cell functions [29]. For example, CRK could control the suppression and activation of natural killer cells [30]. It also could regulate natural killer cell differentiation and expansion during mouse virus infection [31]. DAB2IP is one of the members of Ras GTPase superfamily implicated in the regulation of cell metastasis, apoptosis, and proliferation; it also has been associated with immune cell infiltrates in renal cell carcinoma [32]. LRP8 is an important member of the low-density lipoprotein receptor family and plays a vital role in the synaptic plasticity of brain tissue [33, 34]. RELN gene variants may play an important role in both hippocampal formation and AD pathogenesis [35, 36]. Decrease of RELN expression is an early phenomenon of AD's pathology [37]. STAT5A is indispensable in T regulatory cell development and maintenance and involved in T helper 17 cell differentiation [38]. An increase of STAT5A expression plays a vital role in leukemia development [39]. STAT5A also plays a distinct role in T cell development [40]. CDK5R1 plays an important role during neurodevelopment and is associated with the development of neurodegenerative diseases [41]. The previous study has revealed that CDK5R1 implicated in AD pathogenesis is regulated by the miR-15/107 family of miRNAs, which is anomalously regulated in AD [14]. FYN is an important regulator in neurodegenerative pathways [42]. It has been reported that targeting FYN could rescue memory deficits in an AD mouse model [43]. In our study, we found that the expressions of the seven Reelin pathway-related genes (CRK, DAB2IP, LRP8, RELN, STAT5A, CDK5R1, and FYN) were significantly correlated with some immune cell infiltration levels in AD patients, implying that these genes may play an important role in immune infiltrates of AD.

We also assessed the infiltrating level of immune cells in the two subgroups of AD, which could provide new insight into AD pathogenesis. We found that the proportions of plasma cells, T cell follicular helper, and NK cells activated were significantly lower in the C1 subgroup than the C2 subgroup, whereas the proportions of monocytes, macrophage M2, T cell gamma delta, and dendritic cells activated were higher in the C1 subgroup than the C2 subgroup. AD is a chronic inflammatory disease; a role of the immune response in AD development and progression has been proposed [44, 45]. Abnormal production of inflammatory cytokines by activated NK cells is thought to be partly responsible for the neurodegenerative process of AD [46]. Besides, NK cell activity is negatively correlated with the cognitive status assessed by the analysis of Mini-Mental State Examination score in AD patients [47]. The state of macrophage M2 is typically related to restorative processes of inflammation [48]. It has been demonstrated that macrophage infiltrating the aged brain may be impacted by the inflammatory environment and subsequently influence neuronal health [49, 50]. Macrophage M2 transplantation improves cognitive deficits in the AD model of rats [51]. Monocytes are the major elements in the clearance of amyloid-*β* and play an important role in the development of AD [52, 53]. The previous study has indicated that the blood dendritic cell levels are decreased in AD patients, which is associated with AD progression and severity of depressive symptoms [54]. In our study, significantly different infiltration levels of dendritic cells, monocytes, macrophage M2, and NK cells activated were found in the two subgroups (C1 and C2), implying a possible difference in the pathological process of AD patients. However, there are several limitations in the present study. First, larger clinical sample sizes are needed to verify the CDK5R1 expression. Second, the role of Reelin pathway-related genes in AD should be further investigated in AD-related cell or animal models.

## 5. Conclusion

We identified two CDK5R1-related AD subtypes based on CDK5R1 expression. Our results showed the important role of CDK5R1 in the development and progression of AD and implied that the Reelin pathway-related genes may serve as potential markers for the diagnosis and treatment of AD patients. These findings will help us to further understand the potential function and mechanism of CDK5R1 in AD.

## Figures and Tables

**Table 1:** Basic information of the microarray datasets.

GEO ID	Platform ID	Normal group	AD group	Source	Application
GSE48350	GPL570	173	80	Brain tissue	Analysis
GSE1297	GPL96	9	7	Hippocampal CA1 tissue	Verification
GSE33000	GPL4372	157	310	Prefrontal cortex brain tissue	Verification

**Table 2:** Functional enrichment analysis of the DECRGs.

Ontology	ID	Description	GeneRatio	BgRatio	*p* value	*p*.adjust	*q* value
BP	GO:0050808	Synapse organization	20/194	408/18670	1.02*e*-08	2.97*e*-05	2.52*e*-05
BP	GO:0042391	Regulation of membrane potential	20/194	434/18670	2.84*e*-08	4.15*e*-05	3.53*e*-05
BP	GO:0034765	Regulation of ion transmembrane transport	20/194	483/18670	1.62*e*-07	1.58*e*-04	1.34*e*-04
BP	GO:2001257	Regulation of cation channel activity	12/194	178/18670	3.62*e*-07	2.65*e*-04	2.25*e*-04
BP	GO:1904062	Regulation of cation transmembrane transport	16/194	342/18670	6.05*e*-07	3.54*e*-04	3.01*e*-04
BP	GO:0006836	Neurotransmitter transport	14/194	269/18670	8.93*e*-07	4.35*e*-04	3.70*e*-04
BP	GO:0051216	Cartilage development	12/194	209/18670	1.99*e*-06	8.31*e*-04	7.07*e*-04
BP	GO:0032412	Regulation of ion transmembrane transporter activity	13/194	260/18670	3.45*e*-06	0.001	0.001
BP	GO:0022898	Regulation of transmembrane transporter activity	13/194	268/18670	4.80*e*-06	0.002	0.001
BP	GO:0002062	Chondrocyte differentiation	9/194	123/18670	5.57*e*-06	0.002	0.001
KEGG	hsa04080	Neuroactive ligand-receptor interaction	14/85	341/8076	1.09*e*-05	0.001	0.001
KEGG	hsa05033	Nicotine addiction	5/85	40/8076	5.65*e*-05	0.004	0.004
KEGG	hsa05032	Morphine addiction	6/85	91/8076	3.71*e*-04	0.017	0.016
KEGG	hsa04727	GABAergic synapse	5/85	89/8076	0.002	0.080	0.078

## Data Availability

All data in the present study can be obtained from the corresponding author upon reasonable request.
